# Septoplasty with and without additional sinonasal surgery: postoperative sequelae and the use of prophylactic antibiotics

**DOI:** 10.1007/s00405-021-07113-9

**Published:** 2021-10-15

**Authors:** Ida Kotisalmi, Maija Hytönen, Antti A. Mäkitie, Markus Lilja

**Affiliations:** 1 Department of Otorhinolaryngology-Head and Neck Surgery, University of Helsinki and HUS Helsinki University Hospital, P.O. Box 263, 00029 HUS Helsinki, Finland; 2 Division of Ear, Nose and Throat Diseases, Department of Clinical Sciences, Intervention and Technology, Karolinska Institutet and Karolinska University Hospital, Stockholm, Sweden; 3 Research Program in Systems Oncology, Faculty of Medicine, University of Helsinki, Helsinki, Finland

**Keywords:** Antibiotic prophylaxis, Nasal surgical procedures, Postoperative complications, Rhinosurgery, Septocolumelloplasty, Surgical site infection

## Abstract

**Purpose:**

One of the most common complications after septoplasty is a postoperative infection. We investigated the number of postoperative infections and unplanned postoperative visits (UPV) in septoplasties with and without additional nasal surgery at our institution and evaluated the role of antibiotic prophylaxis.

**Methods:**

We collected data of all consecutive 302 septoplasty or septocolumelloplasty patients operated during the year 2018 at the Department of Otorhinolaryngology-Head and Neck Surgery, HUS Helsinki University Hospital (Helsinki, Finland). Hospital charts were reviewed to record sociodemographic patient characteristics and clinical parameters regarding surgery and follow-up.

**Results:**

Altogether 239 patients (79.1%) received pre- and/or postoperative prophylactic antibiotics and within this group 3.3% developed a postoperative infection. The infection rate in the non-prophylaxis group of 63 patients was 12.7% (*p* = 0.007). When all patients who received postoperative antibiotics were excluded, we found that the infection rate in the preoperative prophylaxis group was 3.8%, as opposed to an infection rate of 12.7% in the non-prophylaxis group (*p* = 0.013). When evaluating septoplasty with additional sinonasal surgery (*n* = 115) the rate of postoperative infection was 3.3% in the prophylaxis group and 16.7% in the non-prophylaxis group (*p* = 0.034). These results show a statistically significant stand-alone effect of preoperative prophylactic antibiotics in preventing postoperative infection in septoplasty, especially regarding additional sinonasal surgery.

**Conclusion:**

The use of preoperative antibiotics as a prophylactic measure diminished statistically significantly the rate of infections and UPVs in septoplasty when all postoperative infections, superficial and mild ones included, were taken into account.

## Introduction

Deviation of the nasal septum causes increased breathing resistance in the nasal passages creating an uncomfortable feeling of inadequate airflow i.e. nasal obstruction. This may further cause decreased quality of life, disturbed sleep, snoring and/or worsen sleep apnoea [1]. Septoplasty is the surgical correction of a deviated nasal septum. The aim of the surgery is to diminish obstruction and thus improve nasal breathing. It is one of the most common ear-, nose- and throat (ENT) surgeries in adults.

After septoplasty there is a risk of both early and late postoperative complications, such as haemorrhage, infection, saddle nose and septal perforation [2–4]. A recent large retrospective study found a 3.1% rate of postoperative septoplasty infections [5], while another reported a rate of 3.3% [6]. At our hospital during the last decades the rate of infections and antibiotic prophylaxis in septoplasties has been repeatedly evaluated [4, 7, 8]. The two first studies reported infection rates of 12% for the late 90’s and 4.2% in the early 2000’s and use of orally administered postoperative antibiotic prophylaxis in 21% and 41% of the cases, respectively [4, 7]. However, the prophylaxis did not appear to be useful in these two retrospective analyses. We then performed a double-blind randomised placebo-controlled study to evaluate the effect of single-dose intravenous cefuroxime given prior to surgery [8]. The infection rate was 2.2% in the antibiotic group and 8.3% in the placebo group (*p* = 0.10). The American Association of Plastic Surgeons has published a recommendation of antibiotic prophylaxis for patients undergoing septoplasty / rhinoplasty [9]. However, the level of evidence in this meta-analysis was low and the grade of recommendation was weak.

The aim of the present study was to investigate the use of antibiotic prophylaxis in every-day clinical practise in septoplasty and septocolumelloplasty with and without additional sinonasal surgery. We were interested in the current indications and implementation of antibiotic prophylaxis at our institution based on the aforementioned studies. Another aim was to assess the effect of antibiotic prophylaxis.

## Materials and methods

### Patients

The study population consisted of all the consecutive patients who had undergone septoplasty or septocolumelloplasty at the Department of Otorhinolaryngology-Head and Neck Surgery, Helsinki University Hospital (Helsinki, Finland) during the year 2018. In this study septocolumelloplasty stands for any septoplasty surgery in which also the anterior part of the cartilage septum (columella or caudal area) is replaced, whereas in septoplasty, the caudal cartilage is left intact. We did not exclude patients who suffered from any other diseases. The study included patients to whom other sinonasal surgery, for example endoscopic sinus surgery or turbinate procedures, had been done during the same operation as septo(columello)plasty. We retrospectively collected data on age, sex, American Society of Anesthesiologist (ASA) score and smoking status prior to the surgery. The patients chosen for septal surgery suffered mainly from nasal obstruction and the diagnosis was confirmed by an ENT surgeon by anterior rhinoscopy and nasal endoscopy. Acoustic rhinometry and rhinomanometry were measured. An institutional study permission was granted for this study.

### Surgical treatment and prophylactic antibiotics

We collected surgical data: septoplasty or septocolumelloplasty, any other sinonasal surgery including radiofrequency ablation of the inferior turbinates (RFAIT), usage of a septal stapler (ENTact, Smith & Nephew, London, UK), nasal packing and/or silicone splints, the type of anaesthesia, duration of the operation and usage of prophylactic antibiotics before and/or after the operation.

### Defining unplanned postoperative visits and surgical site infections

Surgical site infections (SSIs) are postoperative complications involving pathogen infiltration at the surgical wound and occurring within 30 days of the operation. These infections are coupled with symptoms such as fever, purulent drainage, pain, redness and/or localised swelling. They are identified as either superficial, deep incisional or organ based [9–11].

SSIs and any other reasons for unplanned postoperative visits (UPVs) to the treating surgeon within 30 days of the operation were recorded. Patients were considered to have a postoperative SSI if any of the aforementioned symptoms or signs of infection occurred and if the patient had been diagnosed with an SSI by the treating surgeon. The data was collected from patient records and all possible SSIs cases were then reviewed by two rhinologists, and in cases of disagreements furthermore by a third rhinologist, thus determining the SSI cases. UPV was a broader concept, encompassing any symptoms or problems arising postoperatively, e.g. pain, redness or discharge that led to an unplanned follow-up visit but did not fulfil criteria for an SSI.

### Statistical analysis

For analysis of the data, we used SPSS Statistics Version 25 (SPSS, Inc., Chicago, IL, USA, https://www.ibm.com/analytics/spss-statistics-software). The Chi-squared test was used for evaluation of differences in categorical variables between groups. The two-sided *p* value was calculated using either Pearson Chi-squared test or Fisher’s exact test. Fisher’s exact test was used if over 20% of the cells in the Chi-squared test had an expected count of less than 5. The normal distribution of the continuous variables was tested. The Mann–Whitney *U* test was used instead of the *t* test, if an assumption of normality was not fulfilled. The statistical significance level was established at *p* < 0.05.

## Results

### Patient population

The study population consisted of 302 patients with a mean age of 40.5 years (± 14.5 SD; range 8–88) and a sex ratio of 236 males (78.1%) to 66 females (21.9%). Hundred and thirty-three patients were non-smokers, while 94 patients were former smokers, 73 active smokers at the time of the surgery and for two patients smoking status was unknown. There was neither specific information about the extent of smoking habits among the patients, nor could these be defined based on the medical records alone. Detailed information on patients, surgical treatment, and the use of antibiotics in prevention of infection are presented in Table 1.

**Table 1:** Characteristics related to patients and surgical treatment

	Number of patients (%)
Sex	
Female	66 (21.9)
Male	236 (78.1)
Age (median; Q1, Q3)	40; 30, 52
Smoking	
Current	73 (24.2)
Former	94 (31.1)
Never	133 (44.0)
Data missing	2 (0.7)
ASA score	
1	113 (37.4)
2	84 (27.8)
3	30 (9.9)
4	1 (0.3)
Data missing	74 (24.5)
BMI (median; Q1, Q3)	26.2; 23.7, 28.8
Septal surgery	
Septoplasty	198 (65.6)
Septocolumelloplasty	104 (34.4)
Duration of surgery in minutes (median; Q1, Q3)	89.5; 69.8, 111.0
Additional surgery	
Middle turbinate surgery	19 (6.3)
Inferior turbinate surgery; only RFAIT	77 (25.5); 53 (17.5)
Middle meatal surgery	41 (13.6)
Use of specific equipment or materials	
Septal stapler	106 (35.1)
Silicone splints	163 (54.0)
Nasal packing	127 (42.1)
Antibiotic prophylaxis	
Preoperative cefuroxime	229 (75.8)
Preoperative clindamycin	2 (0.7)
Postoperative cefalexin	24 (7.9)
Postoperative doxycycline	2 (0.7)

*ASA score* a physical status classification system by American Society of Anesthesiologists, *BMI* body mass index, *RFAIT* radiofrequency ablation of the inferior turbinates, *Q1* the first quartile, *Q3* the third quartile

### Prophylactic antibiotics

Altogether, 239 patients (79.1%) were given prophylactic antibiotics before and/or after surgery. Solely preoperative prophylactic antibiotics were given to 213 patients (70.5%) 30–60 min prior to incision, according to recent guidelines [10, 12, 13], in the form of an intravenous dose of 1500 mg cefuroxime, and in two cases of cephalosporin allergies 600 mg clindamycin. Solely postoperative antibiotic treatment was given to 8 patients (2.6%), while 18 patients (6.0%) got both pre- and postoperative antibiotics. The postoperative antibiotic prophylaxis consisted of a week-long oral course of cefalexin 500 mg (/250 mg/750 mg three times a day mostly for one week) or doxycycline 100 mg (/150 mg once a day for one week) to be started on the day of the surgery or one day postoperatively. Cefalexin was used in all but two cases, in which doxycycline was used instead. One of these patients had a continuous treatment of doxycycline for chronic rhinosinusitis and in our analysis this case was placed in the postoperative antibiotic prophylaxis group. No adverse effects regarding the use of antibiotics were reported.

### Factors that may affect the use of a prophylactic antibiotic

We examined whether or not the type of procedure (septoplasty vs. septocolumelloplasty, additional surgery and usage of stapler, silicone splints or nasal packing), the type of anaesthesia (local vs. general), age, sex, ASA score, smoking or BMI contributed to the usage of prophylactic antibiotics. We found that antibiotic prophylaxis was used in higher rates in septocolumelloplasty compared with septoplasty (*p* = 0.022). In addition, the use of antibiotic prophylaxis was found to be positively associated with usage of silicone splints (*p* = 0.002), and a longer duration of surgery (*p* < 0.001). We also found antibiotic prophylaxis to be more common in combination with general anaesthesia (*p* = 0.031). Instead, antibiotic prophylaxis was less commonly used in combination with a septal stapler (*p* = 0.041). More detailed data are shown in Table 2.

**Table 2:** Factors that may affect the use of a prophylactic antibiotic

	Antibiotic (%)	*p* value
(A) The proportions of the variables and the statistical differences are presented		
Anaesthesia		
Local	119 (74.4)	
General	120 (84.5)	**0.031**
Sex		
Female	53 (80.3)	
Male	186 (78.8)	0.792
ASA score		
ASA 1–2	161 (81.7)	
ASA 3–4	25 (80.6)	0.885
Smoking		
Never and former	179 (78.9)	
Smoker	58 (79.5)	0.913
Primary surgery		
Septoplasty	149 (75.3)	
Septocolumelloplasty	90 (86.5)	**0.022**
Additional surgery		
No	148 (79.1)	
Yes	91 (79.1)	0.998
RFAIT as additional surgery		
No	183 (79.2)	
Yes	56 (78.9)	0.950
Septal stapler		
No	162 (82.7)	
Yes	77 (72.6)	**0.041**
Silicone splints		
No	99 (71.2)	
Yes	140 (85.9)	**0.002**
Nasal packing		
No	133 (76.0)	
Yes	106 (83.5)	0.115

	No antibiotic	Antibiotic	*p* value
(B) Median (Q1, Q3) of both groups and the statistical differences between groups are presented			
Age	40 (29, 54)	40 (30, 51)	0.742
BMI	24.9 (23.3, 28.9)	26.3 (23.9, 29.1)	0.482
Duration of surgery in minutes	70 (61, 100)	93 (74, 114)	** < 0.001**

Categorical variables are presented in (A) and continuous variables in (B)

*ASA score* a physical status classification system by American Society of Anesthesiologists, *BMI* body mass index, *RFAIT* radiofrequency ablation of the inferior turbinates, *Q1* the first quartile, *Q3* the third quartile

Bold values indicate statistical significance (*p* < 0.05)

### Possible factors underlying postoperative infection and unplanned visits

We analysed whether or not there were any other contributing factors to the occurrence of postoperative infection and unplanned visits within 30 days after the surgery, beyond the usage of antibiotic prophylaxis. We performed statistical analyses on age, sex, ASA score, BMI, smoking status, as well as factors regarding the operation itself, such as the type of procedure (septoplasty vs. septocolumelloplasty, additional surgeries, the usage of a septal stapler, silicone splints or nasal packing, duration of the operation, and the usage of prophylactic antibiotics), and type of anaesthesia. We found that the prophylactic antibiotic statistically significantly reduced both UPVs (*p* = 0.004) and SSIs (*p* = 0.007). We also found that UPVs were less frequent with septocolumelloplasty compared with septoplasty (*p* = 0.048) and that the median length of operation was shorter for patients with UPVs (*p* = 0.045). Furthermore, even if statistical significance was weak, the same trend was observed in the SSI rate. Other statistically significant differences were not found (*p* > 0.05). More detailed data are shown in Table 3. When the same statistical tests were repeated for a group of 213 patients who received solely a preoperative antibiotic, no statistically significant differences were found (*p* > 0.05). Instead, in the group of 63 patients without antibiotic prophylaxis, concurrent inferior turbinate surgery and RFAIT as an additional surgery were associated with a higher incidence of UPVs (*p* = 0.014 and *p* = 0.009, respectively, Fisher’s exact test). No statistically significant differences in the rate of SSIs were observed (Table 4).

**Table 3:** Factors that may affect the rate of unplanned postoperative visits and postoperative infection

	Unplanned postoperative visit (%)	*p* value	Infection (%)	*p* value
(A) The proportions of the variables and the statistical differences are presented				
Anaesthesia				
Local	18 (11.3)		7 (4.4)	
General	14 (9.9)	0.695	9 (6.3)	0.447
Sex				
Female	9 (13.6)		4 (6.1)	
Male	23 (9.7)	0.364	12 (5.1)	0.758
ASA score				
ASA 1–2	20 (10.2)		9 (4.6)	
ASA 3–4	4 (12.9)	0.752	4 (12.9)	0.083
Smoking				
Non and former	26 (11.5)		12 (5.3)	
Smoker	6 (8.2)	0.436	4 (5.5)	1.0
Primary surgery				
Septoplasty	26 (13.1)		14 (7.1)	
Septocolumelloplasty	6 (5.8)	**0.048**	2 (1.9)	0.058
Additional surgery				
No	18 (9.6)		9 (4.8)	
Yes	14 (12.2)	0.485	7 (6.1)	0.631
RFAIT as additional surgery				
No	22 (9.5)		11 (4.8)	
Yes	10 (14.1)	0.275	5 (7.0)	0.543
Septal stapler				
No	23 (11.7)		11 (5.6)	
Yes	9 (8.5)	0.382	5 (4.7)	0.740
Silicone splints				
No	14 (10.1)		9 (6.5)	
Yes	18 (11.0)	0.785	7 (4.3)	0.399
Nasal packing				
No	19 (10.9)		9 (5.1)	
Yes	13 (10.2)	0.863	7 (5.5)	0.888
Prophylactic antibiotic				
No	13 (20.6)		8 (12.7)	
Yes	19 (7.9)	**0.004**	8 (3.3)	**0.007**

	Unplanned postoperative visit		*p* value	Infection		*p* value
	No	Yes		No	Yes	
(B) Median (Q1, Q3) of both group and the statistical differences between groups are presented						
Age	40 (31, 54)	41 (35, 48)	0.815	40 (31, 53)	41 (35, 49)	0.824
BMI	26 (24, 29)	27 (25, 29)	0.322	26 (24, 29)	27 (23, 29)	0.670
Duration	93 (70, 113)	70 (59, 97)	**0.045**	89 (70, 112)	68 (57, 100)	0.109

Categorical variables are presented in (A) and continuous variables in (B). The entire study population of 302 patients was included

*ASA score* a physical status classification system by American Society of Anesthesiologists, *BMI* body mass index, *Duration* duration of surgery in minutes, *RFAIT* radiofrequency ablation of the inferior turbinates, *Q1* the first quartile, *Q3* the third quartile

Bold values indicate statistical significance (*p* < 0.05)

**Table 4:** Factors that may affect the rate of unplanned postoperative visits and postoperative infection when only 63 patients without antibiotic prophylaxis were evaluated

	Unplanned postoperative visit (%)	*p* value	Infection (%)	*p* value
(A) The proportions of the variables and the statistical differences are presented				
Anaesthesia				
Local	7 (17.1)		4 (9.8)	
General	6 (27.3)	0.350	4 (18.2)	0.434
Sex				
Female	5 (38.5)		3 (23.1)	
Male	8 (16.0)	0.119	5 (10.0)	0.345
ASA score				
ASA 1–2	7 (19.4)		5 (13.9)	
ASA 3–4	1 (16.7)	1.0	1 (16.7)	1.0
Smoking				
Non and former	11 (22.9)		7 (14.6)	
Smoker	2 (13.3)	0.716	1 (6.7)	0.6678
Primary surgery				
Septoplasty	11 (22.4)		7 (14.3)	
Septocolumelloplasty	2 (14.3)	0.714	1 (7.1)	0.472
Additional surgery				
No	5 (12.8)		4 (10.3)	
Yes	8 (33.3)	0.062	4 (16.7)	0.467
Middle turbinate surgery				
No	11 (19.3)		7 (12.3)	
Yes	2 (33.3)	0.595	1 (16.7)	0.573
Middle meatal surgery				
No	11 (19.6)		7 (12.5)	
Yes	2 (28.6)	0.627	1 (14.3)	1.0
Inferior turbinate surgery				
No	6 (12.8)		5 (10.6)	
Yes	7 (43.8)	**0.014**	3 (18.8)	0.407
RFAIT as additional surgery				
No	6 (12.5)		5 (10.4)	
Yes	7 (46.7)	**0.009**	3 (20.0)	0.382
Septal stapler				
No	10 (29.4)		6 (17.6)	
Yes	3 (10.3)	0.116	2 (6.9)	0.270
Silicone splints				
No	6 (15.0)		5 (12.5)	
Yes	7 (30.4)	0.129	3 (13.0)	1.0
Nasal packing				
No	7 (16.7)		4 (9.5)	
Yes	6 (28.6)	0.329	4 (19.0)	0.423

	Unplanned postoperative visit		*p* value	Infection		*p* value
	No	Yes		No	Yes	
(B) Median (Q1, Q3) of both group and the statistical differences between groups are presented						
Age	45 (29, 57)	42 (37, 51)	0.622	43 (31, 56)	43 (37, 48)	0.918
BMI	24.8 (23.4, 30.8)	26.5 (22.4, 28.7)	0.854	24.9 (23.4, 29.9)	26.8 (22.1, 28.6)	0.596
Duration	69 (57, 100)	67 (55, 70)	0.203	69 (59, 100)	59 (49, 68)	0.074

Categorical variables are presented in (A) and continuous variables in (B)

*ASA score* a physical status classification system by American Society of Anesthesiologists, *BMI* body mass index, *Duration* duration of surgery in minutes, *RFAIT* radiofrequency ablation of the inferior turbinates, *Q1* the first quartile, *Q3* the third quartile

Bold values indicate statistical significance (*p* < 0.05)

### Unplanned postoperative visits, infections and the effect of prophylactic antibiotics

Among the 302 cases in this study, we recorded altogether 32 UPVs (10.6%) out of which 26 cases were possible SSIs. Upon further critical reviewing 16 cases fulfilled the criteria and were determined as true SSIs (5.3%). All of the infections were classified as superficial SSIs and no deep septal abscesses or organ-based SSIs were found. As presented in Table 5, among 239 patients with any form of prophylactic antibiotic treatment, 7.9% of the cases led to an UPV compared with 20.6% of UPVs in a group of 63 patients that did not get any form of prophylaxis (*p* = 0.004). The percentages of SSIs were 3.3% and 12.7%, respectively (*p* = 0.007). Furthermore, we wanted to examine the stand-alone effect of preoperative antibiotics. Therefore, we excluded from the next analysis all patients who were given postoperative antibiotic treatment. Within the remaining group of 276 patients, 213 patients (77.2%) got preoperative antibiotics. We found that both the percentage of UPVs (*p* = 0.007) and SSIs (*p* = 0.013) were statistically significantly lower in the preoperative antibiotic group compared with the non-prophylaxis group. The independent effect of postoperative prophylaxis and the combination of preoperative and postoperative antibiotic did not reach statistically significant differences compared with the non-prophylaxis group (Table 5).

**Table 5:** Occurrence of unplanned postoperative visits and postoperative infections with and without prophylactic antibiotic treatment

Patients	Preoperative antibiotics	Postoperative antibiotics	Unplanned postoperative visit (%)	*p* value	Postoperative infections (%)	*p* value
63	No	No	13 (20.6)	CG	8 (12.7)	CG
18	Yes	Yes	1 (5.6)	0.175	0	0.189
8	No	Yes	0	0.336	0	0.584
213	Yes	No	18 (8.5)	**0.007**	8 (3.8)	**0.013**
239	Preoperative and/or postoperative antibiotics		19 (7.9)	**0.004**	8 (3.3)	**0.007**

All other groups were compared with CG

*CG* control group

Bold values indicate statistical significance (*p* < 0.05)

### Unplanned postoperative visits, infections and the effect of prophylactic antibiotics in the subgroups with and without additional surgery

We divided study material according to additional surgery into two subgroups: (1) septoplasty alone (2) septoplasty with additional sinonasal surgery (Table 6). In the first subgroup (septoplasty alone), antibiotic prophylaxis had no effect on the number of UPVs. Instead, the percentage of SSIs was three times higher in non-antibiotic group compared to the antibiotic prophylaxis group, but the difference did not reach statistical significance (10.3% vs 3.4%, *p* = 0.092). In the other subgroup (septoplasty with additional sinonasal surgery), antibiotic prophylaxis statistically significantly reduced both the number of UPVs (*p* = 0.002) and SSIs (*p* = 0.0341). All details are presented in Table 6.

**Table 6:** Occurrence of unplanned postoperative visits and postoperative infections with and without prophylactic antibiotic treatment in patients who underwent septoplasty alone (A) and in patients who underwent septoplasty with additional sinonasal surgery (B)

Prophylactic antibiotic	Unplanned postoperative visit (%)	*p* value	Infection (%)	*p* value
(A) Septoplasty alone (*n* = 187)				
No (*n* = 39)	12.8		10.3	
Yes (*n* = 148)	8.8	0.540	3.4	0.092
(B) Septoplasty with additional sinonasal surgery (*n* = 115)				
No (*n* = 24)	33.3		16.7	
Yes (*n* = 91)	6.6	**0.002**	3.3	**0.034**

Bold values indicate statistical significance (*p* < 0.05)

## Discussion

We collected data on all consecutive 302 septoplasty or septocolumelloplasty patients operated during the year 2018. Altogether 115 patients (38.1%) received some kind of additional sinonasal surgery beyond the septoplasty. According to our findings prophylactic antibiotics used in septo(columello)plasties seem to be useful in preventing short-term postoperative complications, such as SSIs or any other reasons resulting in an unplanned visit to the treating surgeon. This especially if patients also require additional surgery, such as RFAIT. More research is required within this field of study to determine our results.

Georgiou et al. [14] reviewed studies on the incidence of postoperative infection in the absence of prophylactic antibiotics and on the effect of prophylactic antibiotic treatment in elective septoplasty. They concluded routine prophylaxis to be unnecessary due to a generally small amount of arising postoperative complications. There are a few other studies that have come to the same conclusion [14–17]. The majority of the analysed study populations in the review by Georgiou et al. were rather small, varying from groups of 35 to 174 patients. This is often the case in studies examining the effects of prophylactic antibiotics in septoplasty [14–16, 18], which automatically makes it difficult to reach proper statistical significance to prove their effect. Two of the studies analysed by Georgiou et al. had considerably larger study populations (1040 and 2000 rhinoplasty patients) and both reported only few postoperative infections (0.48% and 0.6%, respectively), whereas our study presented noticeably higher rates, as high as 5.3%. This shows a great variation in the reported number of postoperative infections in septoplasty, which in turn could be explained by differences in criteria and definition of these postoperative infections in rhino- and septoplasties. We explicitly defined postoperative infections according to published international consensus criteria [9–11]. These same criteria for defining postoperative infection were used by Ariyan et al. [9] who established a consensus statement on evidence-based recommendations for usage of antimicrobial prophylaxis in different types of plastic surgery. They concluded antibiotic prophylaxis to be recommended in clean-contaminated surgery such as septoplasty [9]. They also recognised the difficulties with diagnosing postoperative infections in septoplasty and agreed that this might be a reason for varying rates of infection reported by study groups.

There are a few more studies that have presented low incidences of postoperative complications in septoplasty. In 2005 Caniello et al. reported no postoperative infections within a study population of 35 patients and therefore concluded prophylactic antibiotic treatment to be unnecessary [15]. In another, retrospective study from the 1980’s, Weimert and Yoder presented four postoperative infections (2.3%) in a population of 174 septo- and rhinoplasty patients [14]. In a more recent study Ritter et al. reported no postoperative complications in a study population of 55 septoplasty patients [18]. Whereas, a retrospective study from 2018, as mentioned earlier, reported an incidence of 3.1% of postoperative infection in septoplasty [5], while another study from 2019 presented an infection rate of 3.3% [6]. The rates of SSIs are low but existent, when examining larger study groups.

A previous study at our department on 100 septoplasty patients during the 1990’s reported a postoperative infection rate of 12%, while antibiotic prophylaxis was used in 21% of the cases [7]. Two decades later antibiotic prophylaxis was given to 79.1% of the cases at our centre, and the postoperative infection rate was 5.3%. We can note that usage of antibiotic prophylaxis, especially preoperatively, has increased significantly during these two decades, while the amount of postoperative infections after septoplasty has decreased. In 2011 Lilja et al. [8] reported an infection rate of 5.3% within a study group of 188 patients. Three of these infections were reported as deep incisional SSIs (septal abscesses), all of which occurred within the non-prophylaxis group, whereas, in our current study the postoperative infections consisted of only superficial SSIs. Postoperative infections in septoplasty nowadays seem to be of the milder kind. One reason might be the proper use of antibiotics: a single intravenous dose preoperatively. The total percentage of SSIs, however, has not changed considerably. Could it be that nowadays patients are more sensitive to various postoperative symptoms or any kind of discomfort and therefore, seek medical attention with a lower threshold than before? Even normal postoperative findings can be interpreted as SSIs. These factors, along with different diagnostic criteria, might explain some of the differences in the reported percentage of postoperative infections in septoplasty. Still, we can speculate if our study from 2011 [8] might have been the driving factor for the increased use of prophylactic antibiotics at our department during the last decade.

Even though most of the postoperative infections are indeed mild and only in few cases of the severe kind (abscess, deep incisional or organ based SSI, sepsis, toxic shock syndrome, endocarditis, osteomyelitis, meningitis, cavernous sinus thrombosis), it is important to minimise even these few mild infections that do occur postoperatively. Any arising complications hinder normal healing of the wound and might damage the outcome of the operative treatment and thus it is immensely important to prevent these complications from emerging [19].

In the present study, antibiotic therapy with a prophylactic purpose was used in 79.1% of the cases. We found that usage of antibiotics was statistically significantly more common in septocolumelloplasties, in surgeries of longer duration and if general anaesthesia or silicone splints were used. In general, it seems that surgeons use antibiotic prophylaxis more often in challenging cases as opposed to shorter and simpler ones. All surgeries were performed by a total of 20 surgeons. Therefore, individual variations were possible and even probable, but these were not evaluated any further in this study.

In this study postoperative antibiotic prophylaxis did not turn out to have any statistical significance in diminishing SSIs in septoplasty. A possible contributing factor might be the small number of patients treated with a postoperative antibiotic. According to recent guidelines the prophylactic antibiotic should be administered as a single dose 30–60 min before incision is made to establish the right therapeutic levels at the initiation of the operation, as well as throughout the surgical procedure, but no longer than a few hours after closing the wound [12, 13]. Neither did we find any statistically significant indication that usage of both pre- and postoperative antibiotics should be used to decrease the incidence of SSIs in septoplasty. Usage of both types of antibiotic prophylaxis together would on the other hand possibly and needlessly increase the presence of adverse effects in patients.

When we analysed the material as a whole, we found that an increased rate of UPVs was positively associated with a shorter duration of septoplasty surgery. According to a large systematic review the risk of developing an SSI rises linearly with the duration of the surgery, although notably, the review did not specifically contain any cases of nasal surgery [20]. Our findings seemed to be logically unexpected and further analyses showed that these findings could be explained by a lesser use of antibiotics during shorter surgeries.

In the population of 63 cases without any prophylactic antibiotic, RFAIT as an additional intervention raised statistically significantly rates of UPVs (Table 4). However, the same findings were not observed in the whole material of 302 cases, or in the material of 213 patients all receiving a preoperative single intravenous dose of antibiotic. It appears that the use of antibiotics affected the number of SSIs so strongly that it negated the effect of other variables. Our findings are in line with another study on 5639 patients that concluded turbinectomy, and therefore, RFAIT as additional surgery, to be associated with a higher occurrence of postoperative infection [5]. However, the role of RFAIT as additional surgery should be assessed more. In our study the results may be too biased to allow any further conclusions.

We showed that usage of silicone splints did not increase the rate of SSIs in septoplasty. This is the same outcome as in our previous study [8]. In addition, the use of nasal packing or a stapler was not associated with a higher rate of SSIs. The latter finding correlates with a study by Sainio et al. on 457 patients evaluating the safety of using a stapler in septoplasty [21]. The duration of the use of nasal packing was short in this material, which might explain the absence of any cases of toxic shock syndrome.

The most considerable weakness of this study is its retrospective design. Our study population being rather small with only 302 patients was another limiting factor, although notably many reports on septoplasty have even smaller study populations. On the other hand, a significant strength compared with a prospective trial is the absence of selection bias. Our study group included all septo(columello)plasties performed at our department during the year 2018 and therefore also consisted of patients who had undergone any kind of additional sinonasal surgery beyond the septoplasty itself. This shows a real-world assortment of ENT patients, not a constructed setup as in a prospective study setting. We recognise the bias it generates in regard to the analysis, and this we have taken into account when making our conclusions. We thus believe that the present results are rather generalizable.

## Conclusions

The present study showed that the use of preoperative antibiotic prophylaxis in septo(columello)plasty has increased during the past two decades at the Helsinki University Hospital. Most typically antibiotic prophylaxis was given as a single dose of intravenous antibiotic before the beginning of surgery according to recent international recommendations. The use of preoperative antibiotic treatment as a prophylactic measure statistically significantly diminished the rate of SSIs, and more broadly short-term complications in general, in septoplasty when all SSIs, superficial SSIs included, were taken into account, especially in connection to additional sinonasal surgery.

## Data Availability

Not applicable.
